# Biotransformation of beta-endorphin and possible therapeutic implications

**DOI:** 10.3389/fphar.2014.00018

**Published:** 2014-02-19

**Authors:** Naghmeh H. Asvadi, Michael Morgan, Amitha K. Hewavitharana, P. Nicholas Shaw, Peter J. Cabot

**Affiliations:** School of Pharmacy, The University of QueenslandBrisbane, Queensland, Australia

**Keywords:** peptide, biotransformation, inflammation, opioid, proteomics, pain

Endogenous opioid peptides have been aligned with a diverse array of effects. Their activity is not only attributable to action the three main opioid receptors, mu (MOR), delta (DOR), and kappa (KOR) opioid receptors but their impacts appear to extend to activities at sodium channels, cytokine receptors (Finley et al., 2008), calcium channels and non-specific and partially undefined pharmacological effects inconsistent with G-protein coupled opioid receptor activity.

Of the family of opioid peptides beta-endorphin (BE 1-31) is one of the most prominent and is the prototypical endogenous peptide for the MOR class of opioid receptors and is found within the CNS and the immune system (Cabot et al., 1997). BE 1-31 is derived from pro-opiomelanocortin (POMC) in the cytosol of cell bodies. BE has been shown to possess peripheral and central analgesic activity (Van Den Burg et al., 2001), producing a morphine-like effect by inhibiting the signals of C- and Aδ-fiber activation (Duggan and Fleetwood-Walker, 1993). In addition, BE 1-31 is a non-selective endogenous peptide with the highest affinities for MOR and DOR (Binder et al., 2004), suggesting that the endogenous system is not modulated by specific and selective opioid agonists in isolation.

This concept touches on a new theme evolving in novel therapeutic strategies in the pain field, i.e. the targeting of multiple channels with either one non-selective ligand or a combination of selective ligands to produce effects that are either synergistic or, at a minimum, differential in terms of side effects. This could seemingly point to a multitude of combinations of drugs of both G-protein receptor targeting ligands or extend to those targeting other receptor classes including sodium channels (Su et al., 2002), potassium channels (Welch and Dunlow, 1993) and calcium channels (Smart et al., 1995). The scope of the possible therapeutic targets is immense and, potentially of even greater complexity, is the dose determination for such combinations. Perhaps the answer in part lies in the endogenous opioid system, which is, in essence, the system designed to mediate noxious stimuli as well as interact with the immune system in disease (Figure 1).

**Figure 1:**
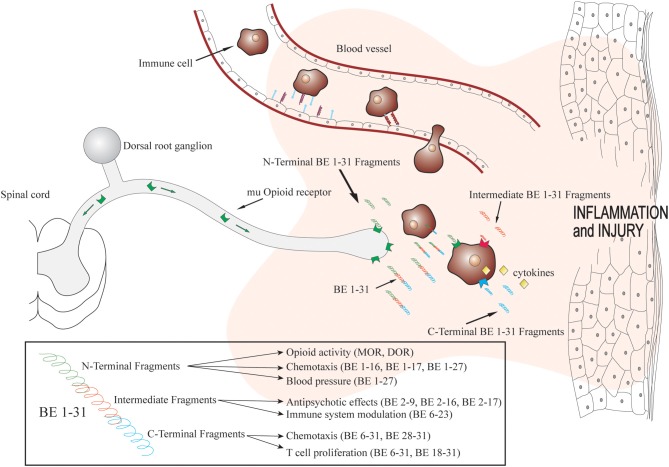
**Biotransformation of beta-endorphin 1–31 within inflamed tissue and fragment actions**. Immune cells containing beta-endorphin migrate to inflamed tissue in a site directed manner. Beta-endorphin is released within the inflammatory mileu and biotransformed rapidly producing fragments with various pharmacological actions. Adapted with permission from Kapitzke et al. (2005).

## Enzymatic processing of beta-endorphin

It is well known that peptides including opioid peptides are susceptible to rapid enzymatic degradation (McKnight et al., 1983). The major peptidases involved in the degradation of opioid peptides are aminopeptidases (Montiel et al., 1997), angiotensin-converting enzyme (ACE), insulin degrading enzyme (Reed et al., 2008), serine peptidases (Sandin et al., 1998), dipeptidyl peptidase III and IV (DPP III, DPP IV) (Sakurada et al., 2003). DPP IV is a serine protease (Shane et al., 1999) and has demonstrated a structural preference for the cleavage of opioid peptides at proline (Augustyns et al., 2005) and is a likely candidate responsible for the cleavage of BE 1–31 producing BE 1–13. Insulin degrading enzyme similarly has been shown to have selective cleaving properties, producing BE 1–17 and BE 1–18 from BE 1–31. In addition, BE 1–19 and BE 20–31 are the likely products of the enzymatic activity of metallo sensitive serine proteases (Sandin et al., 1998). ACE is however has broad peptide cleavage properties and is found widely distributed in many cells (Brownson et al., 1994). Undoubtedly, a major degraditive pathway to non-opioid metabolites will be via aminopeptidases, yet to be demonstrated for BE 1-31 but has been shown to be responsible for cleavage of dynorphin A 1–13 to 2–13 (Müller and Hochhaus, 1995), a conserved region within BE 1–31 and dynorphin A 1–13.

## Inhibiting beta-endorphin biotransformation as a therapeutic strategy

Peptidase inhibition has been investigated as a therapeutic strategy with some success. This is not necessarily a novel approach but it has received a recent resurgence with the development of more selective inhibitors (Schreiter et al., 2012). Certainly, peptidases have been blocked selectively and non-selectively by a number of strategies, e.g., di-isopropyl fluorophosphate and metal ions (Pb^2+^, Hg^2+^, Zn^2+^) are effective inhibitors for DPP IV, albeit di-isopropyl fluorophosphate has been shown to induce compensatory anticholinesterase activity producing tremors due to its irreversible nature. Spinorphin (Tien et al., 2004), which is endogenous factor derived from bovine spinal cord, and its truncated fragment, tynorphin, are both inhibitors of DPP III (Yamamoto et al., 2000). Leupeptin has been shown to block cysteine-containing enzymes and EDTA and phenanthroline inhibit metalloproteases generally (Mentlein, 1999), whilst aminopeptidases are inhibited non-selectively by bestatin without affecting DPP IV (Scornik and Botbol, 2001). A different approach undertaken is to modify the structure of endogenous peptides at specific labile or susceptible bonds. These include the routine modification at Gly^2^ with substitution by D or L Ala^2^ or N-methylation of the Tyrosine^1^, both increasing stability of the peptide by reducing the N-terminal degradation (Hiramatsu et al., 2001). These approaches have been shown to produce long lasting analgesia, thereby suggesting that peptide processing is simply a means to facilitate the degradation of bioactive peptides to their non-pharmacologically active forms. It is likely that this, however, may not be the complete story, opioid peptide fragment and *in-situ* biotransformation may be an integral part of the bodies efforts in addressing disease and pain.

## Biotransformation alteration in disease

A recent study in our laboratory has identified biotransformation fragments of BE 1–31 in rat inflamed tissue (Herath et al., 2012). This study demonstrated that the hydrolytic metabolism of BE 1–31 in homogenized inflamed tissue was faster than in serum and trypsin incubation; similar results have been noted for the processing of dynorphin (the endogenous ligand for KOR) within inflamed tissue homogenates (Morgan et al., 2012). The rate of metabolism of BE 1–31 at pH 5.5 was also higher than the rate of metabolism of BE 1–31 at pH 7.4. These acidic pH values have been shown to be concordant with those found within inflamed tissue (Dray, 1995). In addition, the nature of the biotransformation hydrolysis was altered, BE 1–31 was shown in inflamed tissue homogenates to be most susceptible for hydrolytic degradation at specific amino acid bonds: (Tyr1-Gly2), (Lys9-Ser10), (Leu17-Phe18-Lys19-Asn20), (Lys24-Asn25), (Lys28-Lys29-Gly30-Gln31) (Herath et al., 2012). This is likely to be a consequence of the inflammatory conditions that affect the enzymes independently and specifically (Lin et al., 2001). These results highlight the presence of a unique panel of peptides which would be produced dependent upon the disease state, possessing potentially unique pharmacological properties.

## Biotransformation and opioid activity

Many studies have investigated the pharmacological changes observed following opioid peptide modification and truncation. Deakin et al. showed that the removal of one, two, or four amino acids from the C-terminal of BE 1–31 reduced the analgesic effect of fragments and that the removal of eight amino acids from the N-terminal of BE 1–31 resulted in an absence of analgesic activity (Deakin et al., 1980). Many other studies have provided evidence for the structural necessity of a tyrosine residue at position 1 in BE 1–31 for the retention of analgesic activity. In agreement with this notion, N-acetyl derivatives of BE 1–31 naturally found in the pituitary do not produce opioid activity (Deakin et al., 1980). In addition, a number of studies have demonstrated the C-terminal sequence of BE 1–31determines the potency of opioid peptide in analgesia. Naturally occurring forms of BE 1–31, truncated at the C-terminal, BE 1–28, BE 1–27, and BE 1–26 are found in the pituitary (Zakarian and Smyth, 1982). These compounds are not only ineffective as analgesics but BE 1–27 intra-cerebroventricularly injected into mice has been shown to block the analgesia produced by BE 1–31, with a potency four times greater than that of naloxone - the non-selective opioid antagonist (Hammonds et al., 1984). However, further truncation to BE 1-26 decreased the antagonist effect whilst further reduction of the peptide chain resulted in the complete loss of inhibition of analgesic activity (Nicolas and Choh Hao, 1985). The analgesic potency of further abbreviated forms remains from peptide sequences of BE 1–31 right down to BE 1–4, the overwhelming consequence of the truncation to smaller N-terminal conserved sequences is decreased affinity for MOR, but increased activity at DOR and KOR (Jaba et al., 2007).

## Biotransformation and non-opioid activity

The presence of BE 1–31 in both the neuronal and immune systems indicates that the pharmacological effects of these peptides may extend past those of the management of nociceptive signals. A number of studies have examined potential immune-related mechanisms for BE 1–31 and a variety of truncated forms. Interestingly, effects on human monocyte chemotaxis showed both a lack of requirement for opioid receptor action and the presence of the N-terminal Tyrosine. These effects occurred for a range of truncated forms of BE 1–31 (namely: BE 1–16, BE 1–17, BE 1–27, BE 6–31, BE 28–31) (Sacerdote and Panerai, 1989). Similarly, T cell proliferation was modulated at non-opioid receptors by BE 1–31, BE 6–31, and BE 18–31 (Van Den Bergh et al., 1993). Separate to their immune system effects but aligned with the systemic availability of these peptides, the effects on blood pressure and heart rate in anesthetized rats have also been examined for BE 1–31 and truncated peptides. BE 1–27, shown in previous studies to possess opioid antagonist activity against BE 1–31, and reduced blood pressure to an extent which was similar to that of the effects of the parent molecule, BE 1–31 (Giersbergen et al., 1991). In neurological experiments BE 1–16 and BE 1–17 modulated avoidance behavior and this was not inhibited by naltrexone, an opioid receptor antagonist. The non-opioid peptide fragment BE 2–17 also displayed strong anti-psychotic effects in schizophrenic patients (De Wied, 1979). This non-opioid effect of truncated BE 1–31 was supported in a separate study that showed similar effects with BE 2–16 and BE 2–9 (Van Ree and De Wied, 1982). Furthermore, BE 1–31, when cultured with rat splenocytes, showed suppression of plaque-forming cells (PFC) in response to coculture with sheep red blood cells, not reversed by naloxone (Hemmick and Bidlack, 1989). BE 1–31 has also been shown to interact with protein S in a C-terminal specific manner, implicating BE 1–31 in anticoagulation through antithrombin III (Hildebrand et al., 1989).

## Non-opioid site of action

The search for the sites of action for the non-opioid effects of endogenous opioids has been largely focused on the immune system (Rittner et al., 2008). There is evidence of receptor binding sites for BE 1–31 on a number of immune cells that are not modulated by common analgesics or opioid selective antagonists. There is also a substantial body of evidence for opioids interacting with Toll-like receptors within the immune system (Franchi et al., 2012), with stereo selectivity for the plus isomers of common opioids such as morphine-3-glucuronide (Lewis et al., 2010), naloxone and naltrexone (Hutchinson et al., 2008). These effects have been correlated with modulation of cytokine expression or release, and result in changes that may effect cell proliferation and chemotaxis. Consistent with immune system modulation a non-opioid binding site for BE 1–31 has been demonstrated in immune cells, which would appear to exist in combination with classical opioid receptors and naloxone dependent effects. These sites have been proposed to be activated by restricted sequences of BE 1–31 to BE 6–23 and not modulated by naloxone or alkaloid agonists such as morphine (Kovalitskaya and Navolotskaya, 2011).

## Concluding remarks

Increasing our understanding of the role of beta-endorphin and its biotransformation fragments provides an insight into the complexity of the endogenous opioid system. The current analgesics are targeted at the modulation of analgesia by directly binding to one or more of the opioid receptors, with the analgesic being predominantly designed as a MOP agonist. The above observations would suggest that this is solely one aspect of opioid pharmacology, albeit one that has been explored widely and utilized in therapy. Biotransformation is a process that produces an array of compounds having a plethora of specific actions which contribute to the body's and its biological systems response to disease or injury. Future therapeutic strategies should consider such actions in designing better treatments or disease modulators.
